# Antioxidant Potential of *Artemisia capillaris*, *Portulaca oleracea*, and *Prunella vulgaris* Extracts for Biofabrication of Gold Nanoparticles and Cytotoxicity Assessment

**DOI:** 10.1186/s11671-018-2751-7

**Published:** 2018-10-30

**Authors:** Eun-Young Ahn, You Jeong Lee, Jisu Park, Pusoon Chun, Youmie Park

**Affiliations:** College of Pharmacy and Inje Institute of Pharmaceutical Sciences and Research, Inje University, 197 Inje-ro, Gimhae, Gyeongnam 50834 Republic of Korea

**Keywords:** Gold nanoparticles, Biofabrication, *Artemisia capillaris*, *Portulaca oleracea*, *Prunella vulgaris*, Antioxidant activity, Plant extracts, Cytotoxicity, Protein corona

## Abstract

Three aqueous plant extracts (*Artemisia capillaris*, *Portulaca oleracea*, and *Prunella vulgaris*) were selected for the biofabrication of gold nanoparticles. The antioxidant activities (i.e., free radical scavenging activity, total phenolic content, and reducing power) of the extracts and how these activities affected the biofabrication of gold nanoparticles were investigated. *P. vulgaris* exerted the highest antioxidant activity, followed by *A. capillaris* and then *P. oleracea*. *P. vulgaris* was the most efficient reducing agent in the biofabrication process. Gold nanoparticles biofabricated by *P. vulgaris* (PV-AuNPs) had a maximum surface plasmon resonance of 530 nm with diverse shapes. High-resolution X-ray diffraction analysis showed that the PV-AuNPs had a face-centered cubic structure. The reaction yield was estimated to be 99.3% by inductively coupled plasma optical emission spectroscopy. The hydrodynamic size was determined to be 45 ± 2 nm with a zeta potential of − 13.99 mV. The PV-AuNPs exerted a dose-dependent antioxidant activity. Remarkably, the highest cytotoxicity of the PV-AuNPs was observed against human colorectal adenocarcinoma cells in the absence of fetal bovine serum, while for human pancreas ductal adenocarcinoma cells, the highest cytotoxicity was observed in the presence of fetal bovine serum. This result demonstrates that *P. vulgaris* extract was an efficient reducing agent for biofabrication of gold nanoparticles exerting cytotoxicity against cancer cells.

## Introduction

The interest in metallic nanoparticles has greatly increased owing to their wide array of applications [1]. In particular, gold nanoparticles (AuNPs) have a multitude of valuable applications as drug/gene delivery vehicles, catalysts, imaging agents, and chemical/biological sensors [2]. A variety of chemical and physical methods, such as ion sputtering, reverse micelle, and chemical reduction, are available for fabricating AuNPs. However, these methods are limited by concerns regarding their cost and potential toxicity. The use of plant extracts in the biofabrication (or green synthesis) of AuNPs has advantages over the other methods, which makes it an extremely promising alternative [2]. The biofabrication process is cost-effective and easy to scale up. It utilizes eco-friendly materials and exhibits synergistic activities by combining the activities of two materials (AuNPs and plant extracts). Biofabrication does not introduce noxious chemical reducing agents during the synthesis, which makes the process entirely green. Furthermore, this feature increases the biocompatibility of the resulting AuNPs and increases their stability. Various phytochemicals derived from plant extracts are employed not only as reducing agents but also as capping and stabilizing agents [3].

*Artemisia capillaris* belongs to the genus *Artemisia*, which is one of the largest genera of the family Asteraceae [4]. Since ancient times, *A. capillaris* has been widely used as an herbal medicine owing to its various pharmacological effects, which include antibacterial, anti-inflammatory, antioxidant, and hepatoprotective activities. Previous studies attributed these effects to the active constituents of *A. capillaris*, including capillin, capillene, scoparone, and β-sitosterol [4–6]. *Portulaca oleracea*, an annual succulent in the Portulacaceae family, exhibits various pharmacological effects, such as antibacterial, antifungal, anti-inflammatory, antioxidant, and antiulcerogenic activities. These effects have been demonstrated to result from various active components, including alkaloids, fatty acids, flavonoids, polysaccharides, and terpenoids [7, 8]. *Prunella vulgaris* is a perennial herbaceous plant of the Lamiaceae family. A number of studies have demonstrated the antiviral [9], anticancer [10], immunomodulatory [11], antioxidant [11, 12], and hypoglycemic activities [13] of *P. vulgaris*. The major active components that contribute to these effects are organic acids, phenolic acids, and triterpenoids, including linolenic acid, palmitic acid, *cis-* and *trans-*caffeic acids, rosmarinic acid, oleanolic acid, and ursolic acid [14].

A large gap often exists between in vitro and in vivo outcomes in nanoparticle research due to the “protein corona”. Upon entering a biological environment, the surface of nanoparticles becomes covered with a coating of diverse proteins, which is called the protein corona [15]. The protein corona-coated nanoparticles enter the cell and influence the cellular uptake, cytotoxicity, biodistribution, excretion, cell response, drug release, and therapeutic efficiency [16]. Interestingly, the physicochemical properties of metallic nanoparticles, such as their shape, size, surface charge, and surface roughness, profoundly affect serum protein adsorption when forming the protein corona [17]. Shannahan and coworkers reported that the formation of a protein corona on silver nanoparticles (AgNPs) mediates the cellular toxicity against rat lung epithelial cell and rat aortic endothelial cells *via* scavenger receptors [18]. Serum albumin affects hydrogel-embedded colloidal AgNPs by reducing their antibacterial and cytotoxic effects [19].

In the present report, we assessed the antioxidant activities, including the free radical scavenging activity, total phenolic content, and reducing power, of the aqueous extracts of *A. capillaris*, *P. oleracea*, and *P. vulgaris*. Subsequently, the biofabrication of AuNPs using these three extracts was conducted to investigate how the antioxidant activities of the extracts affect the color of the colloidal solution and the shape of the surface plasmon resonance (SPR) band of the AuNPs. Hereafter, the biofabricated AuNPs are named after the scientific name of each plant: the AuNPs obtained using the extract of *A. capillaris* are referred to as AC-AuNPs, the AuNPs obtained using the extract of *P. oleracea* are referred to as PO-AuNPs, and the AuNPs obtained using the extract of *P. vulgaris* are referred to as PV-AuNPs. The antioxidant activity of the *P. vulgaris* extract was the highest; thus, we characterized the PV-AuNPs by various spectroscopic and microscopic methods, including UV-visible spectrophotometry, high-resolution transmission electron microscopy (HR-TEM), high-resolution X-ray diffraction (HR-XRD), and inductively coupled plasma optical emission spectroscopy (ICP-OES). The hydrodynamic size of the PV-AuNPs was measured by dynamic light scattering (DLS) along with the zeta potential. Furthermore, we examined the antioxidant activity of the PV-AuNPs by monitoring the 2,2-diphenyl-1-picrylhydrazyl (DPPH) radical scavenging activity. The cytotoxicity against three cancer cells was investigated by a water-soluble tetrazolium (WST) assay either in the presence or absence of fetal bovine serum (FBS) in cell culture. The following cancer cells were used: human colorectal adenocarcinoma (HT-29), human breast adenocarcinoma cell (MDA-MB-231), and human pancreas ductal adenocarcinoma cell (PANC-1).

## Methods

### Materials

Potassium gold(III) chloride (KAuCl_4_), butylated hydroxytoluene (BHT), sodium phosphate monobasic, sodium phosphate dibasic, trichloroacetic acid, Folin-Ciocalteu’s phenol reagent, sodium carbonate, gallic acid, potassium sodium tartrate, sodium bicarbonate, sulfuric acid, 2,2′-azino-bis-3-ethylbenzthiazoline-6-sulphonic acid (ABTS), potassium persulfate, and d-(+)-glucose were purchased from Sigma-Aldrich (St. Louis, MO, USA). DPPH was purchased from Alfa Aesar (Ward Hill, MA, USA). Potassium hexacyanoferrate(III) was obtained from Wako (Osaka, Japan). Sodium sulfate, hexa-ammonium heptamolybdate tetrahydrate, and disodium hydrogen arsenate heptahydrate were purchased from Junsei (Tokyo, Japan). Iron(III) chloride was obtained from Duksan (Gyeonggi, Republic of Korea), and copper sulfate pentahydrate was obtained from Deajeng (Gyeonggi, Republic of Korea). The other reagents were of analytical grade and used as received. High-glucose Dulbecco’s modified Eagle’s medium (DMEM), penicillin-streptomycin (10,000 U/mL), trypsin-EDTA (0.5%, without phenol red), and phosphate-buffered saline (PBS) were purchased from Gibco (Thermo Fisher Scientific, MA, USA). FBS was obtained from GE Healthcare HyClone™ (Victoria, Australia).

### Instruments

A Shimadzu UV-2600 spectrophotometer was utilized to acquire UV-visible spectra and observe SPR (Shimadzu Corporation, Kyoto, Japan). Information regarding the particle size and shape was obtained from HR-TEM images. To acquire HR-TEM images, a JEM-2100F instrument operating at 200 kV was utilized (JEOL, Tokyo, Japan). A colloidal solution of PV-AuNPs was loaded on a carbon-coated copper grid (carbon type-B, 300 mesh, Ted Pella, Redding, CA, USA). The sample-loaded grid was air-dried at ambient temperature. A Rigaku Miniflex diffractometer (40 kV, 30 mA) was used to acquire the HR-XRD pattern with a CuKα radiation source (*λ* = 1.54056 Å) in the range of 10º to 80° (2*θ* scale) (Rigaku, Japan). A FD5505 freeze dryer was utilized to prepare powdered samples for HR-XRD analysis (Il Shin Bio, Seoul, Republic of Korea). To estimate the reaction yield, ICP-OES was conducted on a Perkin Elmer Optima 8300 instrument (Waltham, MA, USA). Centrifugation was conducted (18,500*g* force, 7 h, 18 °C), and the supernatant was collected. Two samples, i.e., the colloidal PV-AuNP solution and the supernatant, were subjected to ICP-OES analysis. An Eppendorf 5424R centrifuge was utilized for centrifugation (Eppendorf AG, Hamburg, Germany). Hydrodynamic size and zeta potentials were measured by the NanoBrook 90 Plus Zeta analyzer (Brookhaven Instruments Corporation, Holtsville, NY, USA).

### Preparation of the Aqueous Extracts of *A. capillaris*, *P. oleracea*, and *P. vulgaris*

The dried aerial parts of *P. vulgaris* and *A. capillaris*, including the flowers and leaves, were purchased from Omniherb (Daegu, Republic of Korea), and *P. oleracea* was obtained from Handsherb (Youngchun, Republic of Korea). Aqueous extracts of the aerial parts of each plant were prepared by sonication (WUC-A22H, Daihan Scientific Co. Ltd., Republic of Korea). For each plant, 100 g of the chopped plant was extracted three times with deionized water (1000 mL). The extraction procedure comprised sonication of the plants at 25 °C for 3 h. The aqueous extract was filtered, and the filtrate was lyophilized in a vacuum freeze dryer (FD8518, IlShinBioBase Co. Ltd., Republic of Korea). Finally, the extract powder of each plant was obtained and stored at − 20 °C prior to use.

### DPPH Radical Scavenging Activity

In each well of a 96-well microplate, 20 μL of each extract was mixed with a DPPH radical solution in ethanol (0.1 mM, 180 μL) to give several final concentrations of the extract (31.25 μg/mL, 62.5 μg/mL, 125 μg/mL, and 250 μg/mL). The plate was incubated in the dark for 30 min at ambient temperature, and then, the absorbance of each well was measured at 517 nm using a multi-detection reader (Synergy HT, BioTek Instruments, Winooski, VT, USA). BHT was used as a standard. All experiments were performed in triplicate. The absorbance of the AuNPs was subtracted from the absorbance of each sample to eliminate the intrinsic absorbance of the AuNPs at 517 nm.

### ABTS Radical Scavenging Activity

The ABTS radical cation was prepared by the reaction of a 1:1 (*v/v*) mixture of a 7.4 mM ABTS solution and a 2.6 mM potassium persulfate solution for 24 h in the dark at ambient temperature. The ABTS radical solution was diluted with ethanol to an absorbance of 0.74 at 730 nm for measurements. In each well of a 96-well microplate, the ABTS radical solution in ethanol (180 μL) was added to 20 μL of each extract (final concentrations of the extract, 15.63 μg/mL, 31.25 μg/mL, 62.5 μg/mL, and 125 μg/mL). After incubation for 10 min in the dark at ambient temperature, the absorbance of each well was measured at 730 nm using a multi-detection reader. A solution of vitamin C was prepared as a standard. All experiments were performed in triplicate.

### Reducing Power

Equal volumes (500 μL) of various concentrations of the extracts were mixed with 0.2 M phosphate buffer (pH 6.6, 500 μL) and 1% potassium hexacyanoferrate(III) (500 μL). The final concentrations of the extracts were 52.1 μg/mL, 104.2 μg/mL, 208.3 μg/mL, and 416.7 μg/mL. Each mixture was incubated at 50 °C. After 20 min, trichloroacetic acid (10%, 500 μL) was added to the mixture, followed by centrifugation at 3400*g* force for 10 min. Then, an iron chloride solution (0.1%, 100 μL) was added to 500 μL of the supernatant. After 10 min, the absorbance of the reaction mixture was measured at 700 nm using a multi-detection reader. BHT was used as a standard. All experiments were performed in triplicate.

### Total Phenolic Content

The relationship between the antioxidant activity and the content of phenolic compounds was investigated. Twenty microliters of the extract was mixed with 100 μL of Folin-Ciocalteu’s reagent (diluted 10 times with deionized water) and 80 μL of sodium carbonate (7.5%, *w/v*). The reaction was performed in the dark at ambient temperature. After 30 min, the absorbance at 765 nm was measured, and the total phenolic content was determined using a calibration curve constructed with gallic acid. All determinations were performed in triplicate.

### Biofabrication of the PO-AuNPs, AC-AuNPs, and PV-AuNPs

Two stock solutions were prepared prior to the biofabrication reactions: potassium gold(III) chloride (10 mM) and the extract (2%, *w/v*). The stock solution of each extract (2%) was centrifuged (18,500*g* force, 30 min, 18 °C). The supernatant was collected and utilized for the biofabrication reaction. The final concentrations were adjusted to 0.5 mM potassium gold(III) chloride and 0.05% (*w/v*) extract in a 2 mL glass vial for the biofabrication of AuNPs. After mixing the Au salts with the extract, incubation was performed at 37 °C in a dry oven for 5 h. Then, UV-visible spectra were acquired in the range of 300~800 nm.

### Cell Culture

Three cancer cells (HT-29, PANC-1, and MDA-MB-231) were purchased from the Korean Cell Line Bank (Seoul, Republic of Korea). Cells were grown in high-glucose DMEM supplemented with 10% FBS, penicillin (100 units/mL), and streptomycin (100 μg/mL). Cells were cultured at 37 °C (supplied 5% CO_2_) in an incubator and maintained approximately 80% confluence prior to trypsinization.

### Cytotoxicity

The EZ-CYTOX kit from DoGenBio (Gyonggi, Republic of Korea) was used for the WST assay to assess the cytotoxicity against cancer cells. The cells were seeded in 96-well plates at a density of 5.0 × 10^3^ cells/well. Incubation was performed for 24 h in a 37 °C incubator under a CO_2_ (5%) atmosphere. After incubation, the media was discarded. Then, different concentrations of PV-AuNPs (0, 29.9, 59.8, 119.6, 239.2, and 478.3 μg/mL based on the Au concentration measured by ICP-OES analysis) were added to the cells with the culture medium in either the presence or absence of FBS. After another 24 h of incubation at 37 °C in the incubator, the EZ-CYTOX reagent (10 μL) was added, and the cells were incubated in a CO_2_ incubator for an additional 1 h. The absorbance was measured at 450 nm using a Cytation hybrid multimode reader (BioTek Instruments, Winooski, VT, USA). The intrinsic absorbance of the PV-AuNPs interfered with the measurement of the cytotoxicity at 450 nm. Therefore, the background absorbance of the PV-AuNPs was subtracted from the absorbance of each data point measured at 450 nm. The background absorbance of the PV-AuNPs was measured in WST-free conditions. In addition, the same volume of de-ionized water was utilized as a control instead of the PV-AuNPs.

### Statistical Analysis

All experiments were performed in triplicate, and the results are presented as the mean ± standard error (SE). The significance of the differences was investigated using ANOVA (one-way analysis of variance) followed by Tukey’s multiple comparison test or the Newman-Keuls multiple comparison test. Statistical analyses were computed using GraphPad Software (GraphPad Software version 5.02, San Diego, CA, USA). The results were considered statistically significant for values of *p* < 0.05.

## Results and Discussion

### Antioxidant Activities of the Extracts

**Fig. 1:**
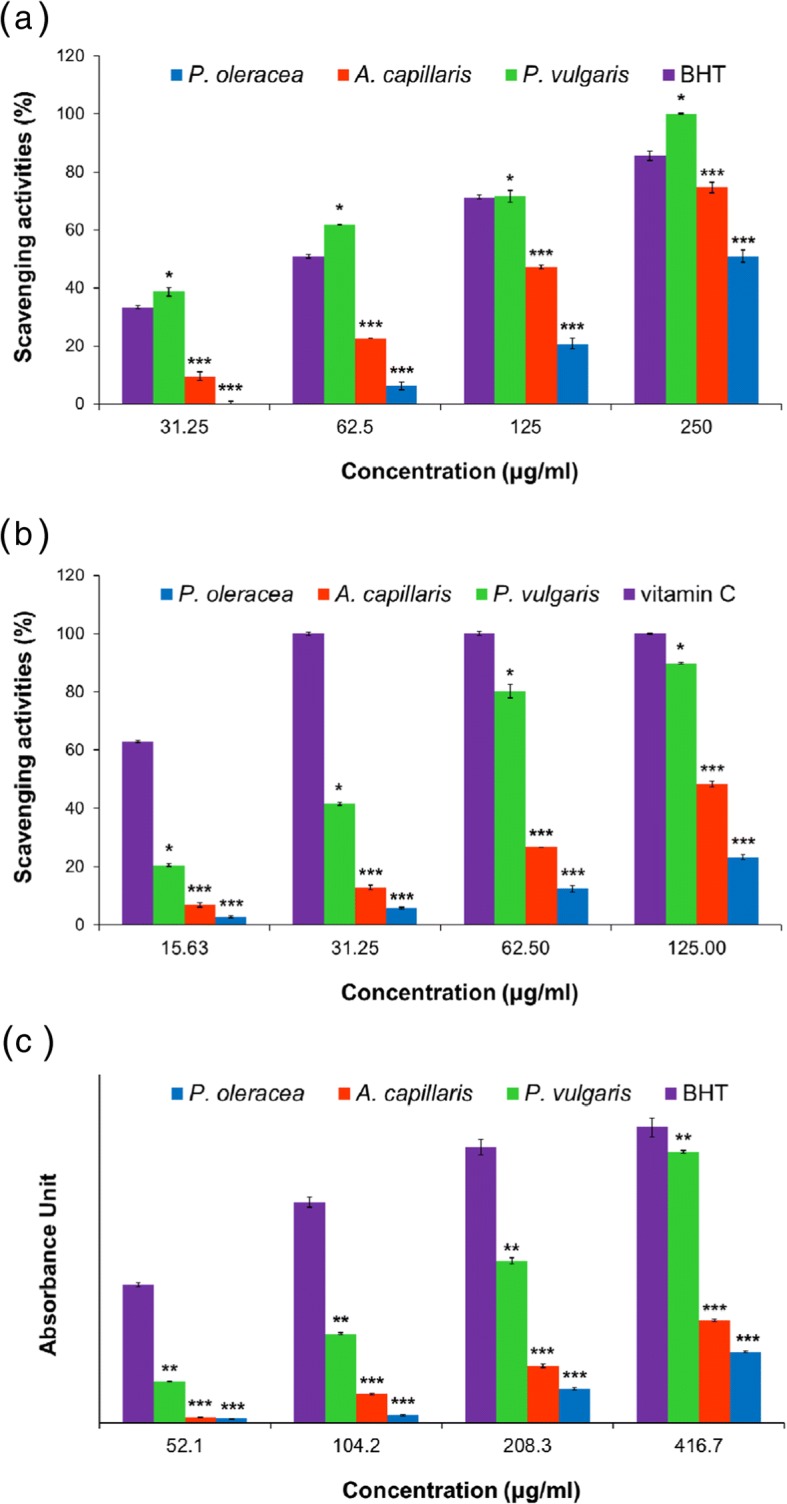
DPPH and ABTS radical scavenging activities and reducing power of the aqueous extracts of *A. capillaris*, *P. oleracea*, and *P. vulgaris*. **a** DPPH radical scavenging activities. **b** ABTS radical scavenging activities. **c** Reducing power of the extracts. The data are presented as the mean ± SEM. The asterisks indicate the significance of difference versus the standard (BHT or vitamin C): *p < 0.05, **p < 0.01, ***p < 0.001. The results are representative of three independent experiments

Aqueous extracts of the aerial parts of each plant were obtained by sonication. The extraction yields of *A. capillaris*, *P. oleracea*, and *P. vulgaris* were 14.1%, 39.12%, and 28.6%, respectively. The highest extraction yield was shown in *P. oleracea*, followed by *P. vulgaris* and lastly by *A. capillaris*. To assess the antioxidant activities of the extracts, we measured the free radical scavenging activity, reducing power, and total phenolic content. The free radical scavenging activities of the extracts were determined by DPPH and ABTS assays. DPPH is widely used to assess the antioxidant activity of phenolic compounds because the DPPH radical is a stable free radical that loses its absorbance intensity when it is reduced by antioxidants. The ABTS assay measures the ability of an antioxidant to scavenge the radicals that are generated by the reaction of a strong oxidizing agent with ABTS. The reduction in the absorbance of the ABTS radical by antioxidants with hydrogen-donating properties is assessed. The aqueous extracts exhibited scavenging activities in a dose-dependent manner. At the concentrations tested (15.63 μg/mL, 31.25 μg/mL, 62.5 μg/mL, 125 μg/mL, and 250 μg/mL), the extract of *P. vulgaris* (IC_50_ 50.35 ± 1.22 μg/mL against DPPH radical; IC_50_ 38.6 ± 0.44 μg/mL against ABTS radical) exhibited the most potent activities against the DPPH and ABTS radicals followed by the extract of *A. capillaris* (IC_50_ 156.72 ± 0.97 μg/mL against DPPH radical; IC_50_ 147.28 ± 2.95 μg/mL against ABTS radical) and then the extract of *P. oleracea* (IC_50_ 247.33 ± 1.57 μg/mL against DPPH radical; IC_50_ 305.54 ± 4.86 μg/mL against ABTS radical). Notably, *P. vulgaris* showed a more potent DPPH free radical scavenging activity than BHT (IC_50_ 71.37 ± 0.84 μg/mL), which was used as a standard (Table 1 and Fig. 1a, b). The extract of *P. vulgaris* demonstrated the highest DPPH and ABTS radical scavenging activities among the three extracts. This result implied that possibly more antioxidant compounds would exist in *P. vulgaris* than in *A. capillaris* and *P. oleracea.*

**Table 1:** Antioxidant activity of *A. capillaris*, *P. oleracea*, and *P. vulgaris* extracts

	IC_50_ against DPPH radical, μg/mL	IC_50_ against ABTS radical, μg/mL	Reducing power; concentration at absorbance (0.7), μg/mL	Total phenolic content, mg GAE/g
*A. capillaris*	156.72 ± 0.97	147.28 ± 2.95	235.38	39.88 ± 4.55
*P. oleracea*	247.33 ± 1.57	305.54 ± 4.86	396.16	18.32 ± 2.76
*P. vulgaris*	50.35 ± 1.22	38.6 ± 0.44	162.98	90.53 ± 6.81
Vitamin C	ND	12.15 ± 0.05	ND	ND
BHT	71.37 ± 0.84	ND	94.36	ND

*ND* not determined

We also examined the antioxidant activities of the extracts *via* a reducing power assay. In the presence of antioxidants, potassium ferricyanide (Fe^3+^) is converted to potassium ferrocyanide (Fe^2+^), which reacts with ferric chloride to form a ferric-ferrous complex. The ferric-ferrous complex has a maximum absorbance at 700 nm, which can be used to evaluate the reducing power of an antioxidant. In the current report, the reducing power is expressed as the concentration (μg/mL) of each extract that gave an absorbance of 0.7 at 700 nm. As shown in Fig. 1c, *P. vulgaris* (162.98 μg/mL) exerted a remarkably higher reducing power than *A. capillaris* (235.38 μg/mL) and *P. oleracea* (396.16 μg/mL). As mentioned previously, the results of reducing power also indicated that possibly more antioxidant compounds would exist in *P. vulgaris* than in *A. capillaris* and *P. oleracea.*

The total phenolic content of each extract was also investigated. It is well known that phenolic compounds are potential antioxidants and possess the ability to scavenge radicals owing to their hydroxyl groups. Thus, the antioxidant activity of a plant extract can correlate well to its phenolic content. A standard calibration curve was constructed using gallic acid, and this curve showed a linear relationship (*y* = 6.0617*x* + 0.1293, *r*^2^ = 0.9983). Using the standard curve, the total phenolic content was calculated and is expressed as milligrams of gallic acid equivalent (GAE) per gram of extract. The total phenolic contents of the extracts were 90.53 ± 6.81 mg GAE/g for *P. vulgaris*, 39.88 ± 4.55 mg GAE/g for *A. capillaris*, and 18.32 ± 2.76 mg GAE/g for *P. oleracea*. The amount of total phenolic contents was the highest in *P. vulgaris* among the three extracts.

There was a positive correlation between the total phenolic contents and the ability of the extracts to scavenge radicals or their reducing power. Taken together, the aqueous extracts of *A. capillaris*, *P. oleracea*, and *P. vulgaris* exhibited significant antioxidant activities. In particular, the extract of *P. vulgaris* showed the largest radical scavenging activity against both DPPH and ABTS radicals, the strongest reducing power, and the highest total phenolic content, implying that the extract of *P. vulgaris* would exert the strongest activity as a reducing agent for the biofabrication of AuNPs. These interesting results of biofabrication of AuNPs using the three extracts will be discussed in the following section.

### Biofabrication of AC-AuNPs, PO-AuNPs, and PV-AuNPs

We have proposed that the antioxidant activity of the extract strongly affects the biofabrication process of AuNPs. Since the antioxidant activity is a major driving force in the reduction of Au salts to AuNPs, *P. vulgaris*, which has the highest antioxidant activity, is capable of being the most efficient reducing agent. The biofabrication process was followed by UV-visible spectrophotometry, as AuNPs possess a distinctive SPR in the visible-near infrared wavelength range. The biofabrication of AuNPs was performed by mixing Au salts with each extract solution. Each extract contained diverse phytochemicals that are very efficient for the reduction of Au salts to AuNPs.

**Fig. 2:**
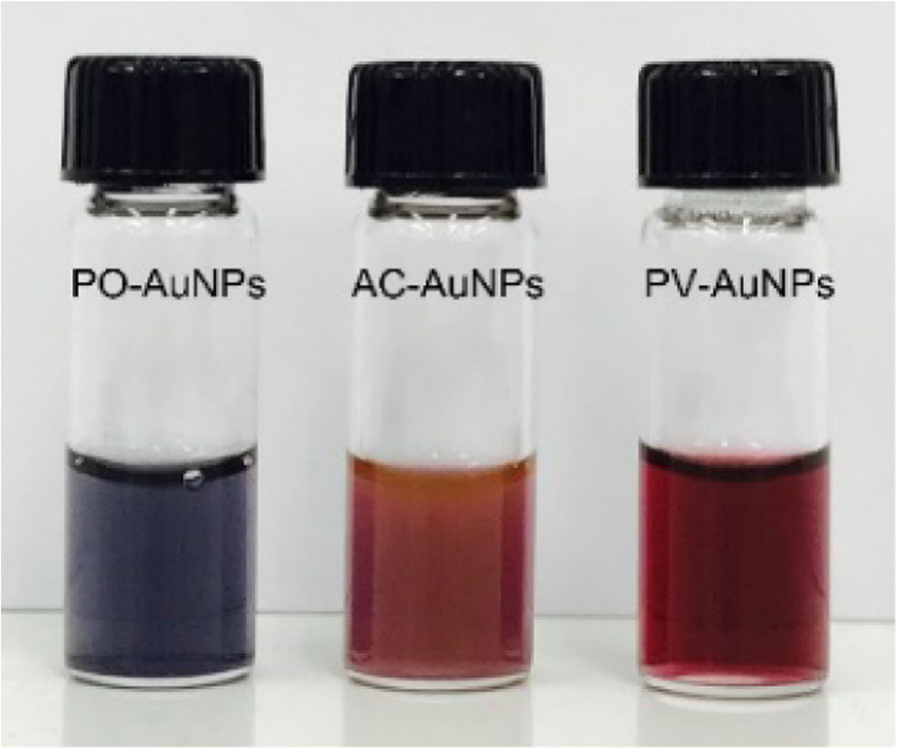
Digital photographs of PO-AuNPs, AC-AuNPs, and PV-AuNPs

**Fig. 3:**
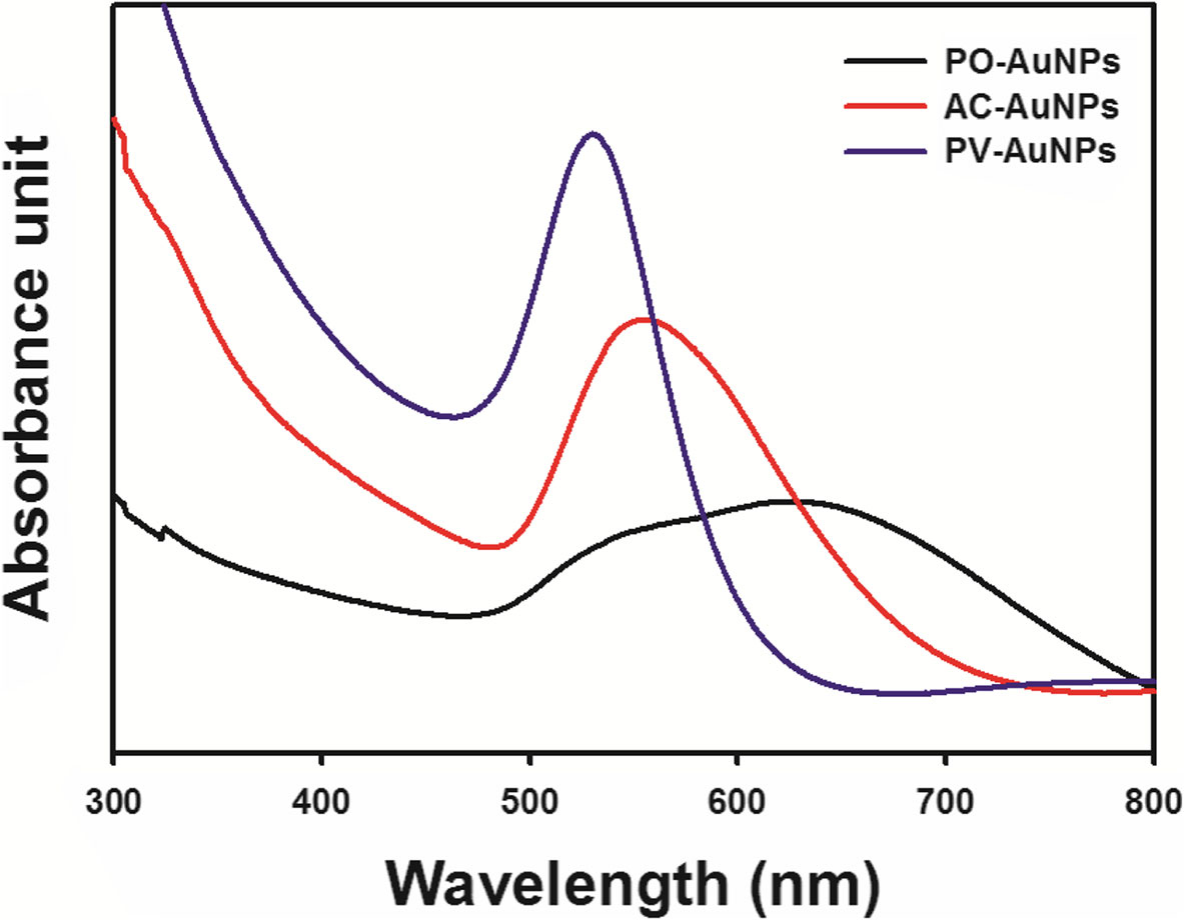
UV-visible spectra of PO-AuNPs (black line), AC-AuNPs (red line), and PV-AuNPs (blue line)

The distinct SPR band of AuNPs resulted in changes in the color of the initially pale-yellow solution to dark blue for the PO-AuNPs, fluorescent brown for the AC-AuNPs, and wine-red for the PV-AuNPs (Fig. 2). UV-visible spectra were recorded after incubation at 37 °C in a dry oven for 5 h to observe the distinct SPR band of each set of AuNPs (Fig. 3). The maximum absorbance was observed at 530 nm for the PV-AuNPs and at 555 nm for the AC-AuNPs. In the case of the PO-AuNPs, a broad SPR band was observed from 500 to 700 nm. The three extracts generated AuNPs with a characteristic SPR band in UV-visible spectra. Each color change together with the distinct SPR band clearly supported the successful biofabrication of AuNPs with each extract. We proposed that the three factors related to the antioxidant activities (free radical scavenging activity, total phenolic content, and reducing power) affect AuNP biofabrication. The extract of *P. vulgaris* scored the highest for all three factors, followed by *A. capillaris* and lastly by *P. oleracea*. Interestingly, the PO-AuNPs were shown to aggregate after storage at ambient temperature (25 °C) for 30 min. This result suggested that the PO-AuNPs were the most unstable, as the antioxidant activity, amount of total phenolic compounds, and reducing power of *P. oleracea* were the lowest among these three extracts. In contrast, the PV-AuNPs showed the highest absorbance and a transparent wine-red solution. The absorbance of the AC-AuNPs was between that of the PO-AuNPs and the PV-AuNPs. Therefore, based on these observations, it was determined that higher free radical scavenging activity, total phenolic content, and reducing power of the *P. vulgaris* extract resulted in the synthesis of more stable AuNPs compared to the AuNPs produced by the extracts of *A. capillaris* and *P. oleracea*. Collectively, the maximum SPR bands showed bathochromic and hypochromic shifts with decreasing antioxidant activity of the extract. Furthermore, the shape of the SPR band tended to broaden with the decrease of antioxidant activity. Accordingly, the PV-AuNPs were further characterized by HR-TEM, HR-XRD, hydrodynamic size and zeta potential measurements. To determine the reaction yield, ICP-OES analysis was conducted. The antioxidant activity and cytotoxicity in the presence/absence of FBS were further evaluated against cancer cells.

### HR-TEM Images of the PV-AuNPs

**Fig. 4:**
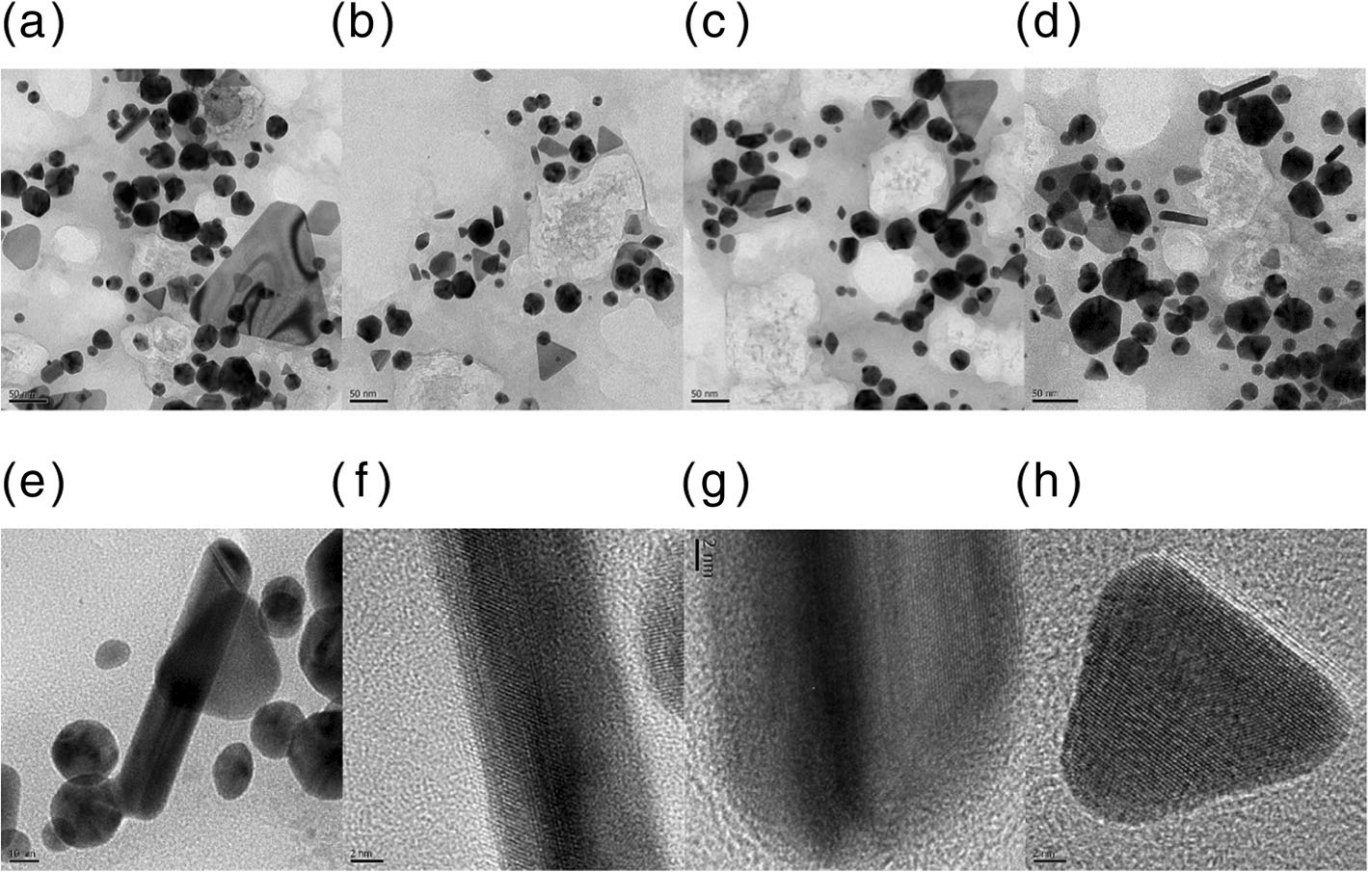
HR-TEM Images of PV-AuNPs. The scale bar represents **a** 50 nm, **b** 50 nm, **c** 50 nm, **d** 50 nm, **e** 10 nm, **f** 2 nm, **g** 2 nm, and **h** 2 nm

**Fig. 5:**
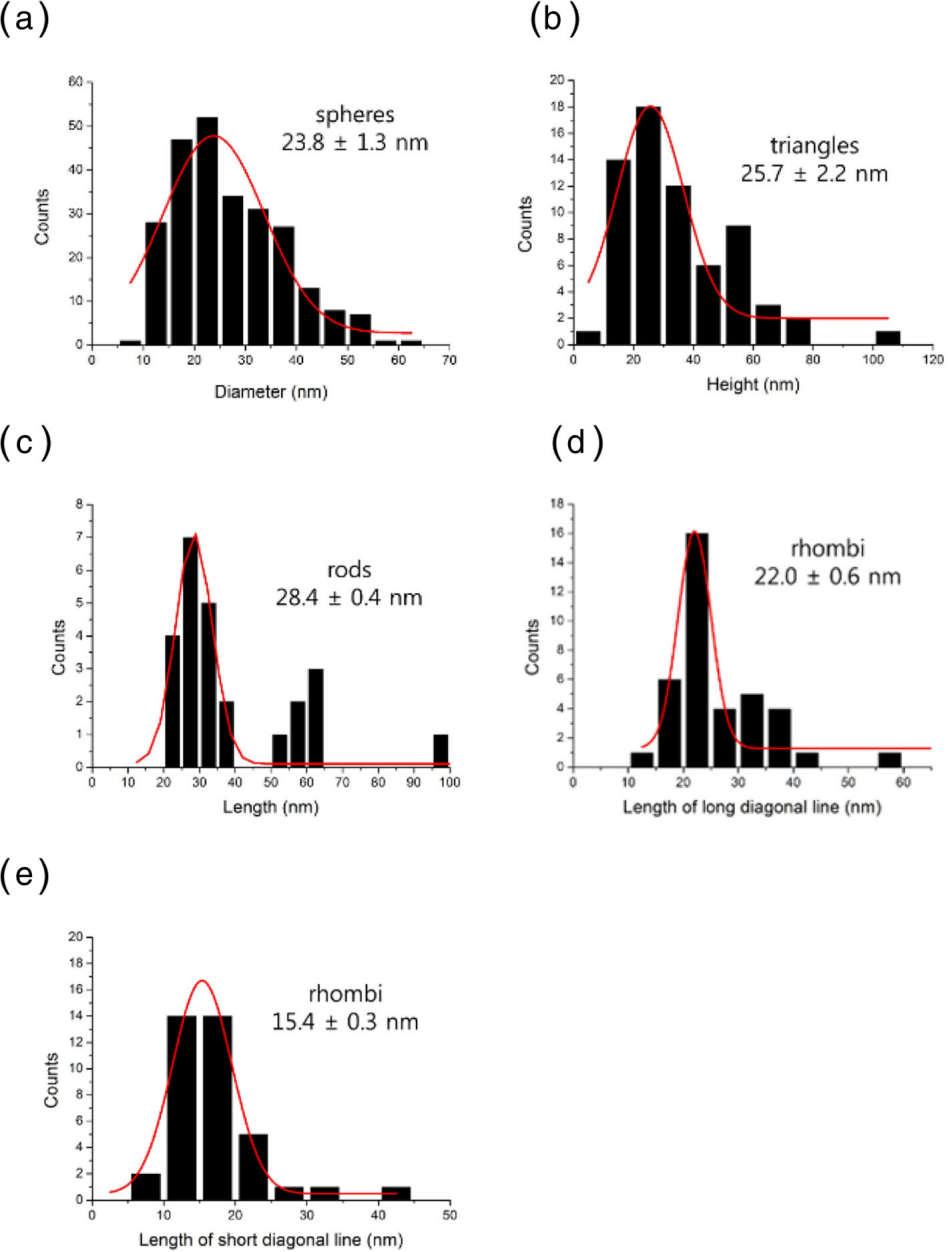
Size histograms of PV-AuNPs. **a** The diameter of spheres. **b** The height of equilateral triangles. **c** The length of rods. **d** The length (long diagonal line) of rhombi. **e** The length (short diagonal line) of rhombi

Microscopic techniques are the most effective tools for visualizing nanoparticles. Along with HR-TEM, scanning electron microscopy and atomic force microscopy are commonly utilized to determine the size, morphology, topography, and two-dimensional/three-dimensional structures. The size and morphology of the PV-AuNPs were investigated with HR-TEM. As shown in Fig. 4, various shapes, including spheres, triangles, rods, rhombi, and hexagons, were observed (Fig. 4a~e). The observation of lattice structures in a rod (Fig. 4f, g) and a triangle (Fig. 4h) clearly demonstrated that the PV-AuNPs were crystalline in nature. This result was strongly supported by the subsequent HR-XRD analysis results. The size of each particle shape was measured from the HR-TEM images, and the resulting size histograms are illustrated in Fig. 5. All histograms showed a Gaussian distribution. Discrete nanoparticles in the images were randomly selected to obtain an average size of each shape. The diameter of the spheres was determined to be 23.8 ± 1.3 nm from 250 nanoparticles (Fig. 5a). Interestingly, equilateral triangles were produced; thus, we measured the heights of 64 nanoparticles (25.7 ± 2.2 nm, Fig. 5b). Twenty-seven rod-shaped nanoparticles were randomly chosen to determine the average length (28.4 ± 0.4 nm, Fig. 5c). The average aspect ratio, defined as the length divided by the width, of the rods was determined to be 2.4. For the rhombus shape, 38 rhombi were randomly selected in the images. As shown in Fig. 5d, e, the average lengths of the long and short diagonal lines were measured to be 22.0 ± 0.6 nm and 15.4 ± 0.3 nm, respectively. Diversely shaped PV-AuNPs possessed the size of less than 30 nm, and the sizes were compared with the hydrodynamic size in the following section.

### Hydrodynamic Size and Zeta Potential of the PV-AuNPs

**Fig. 6:**
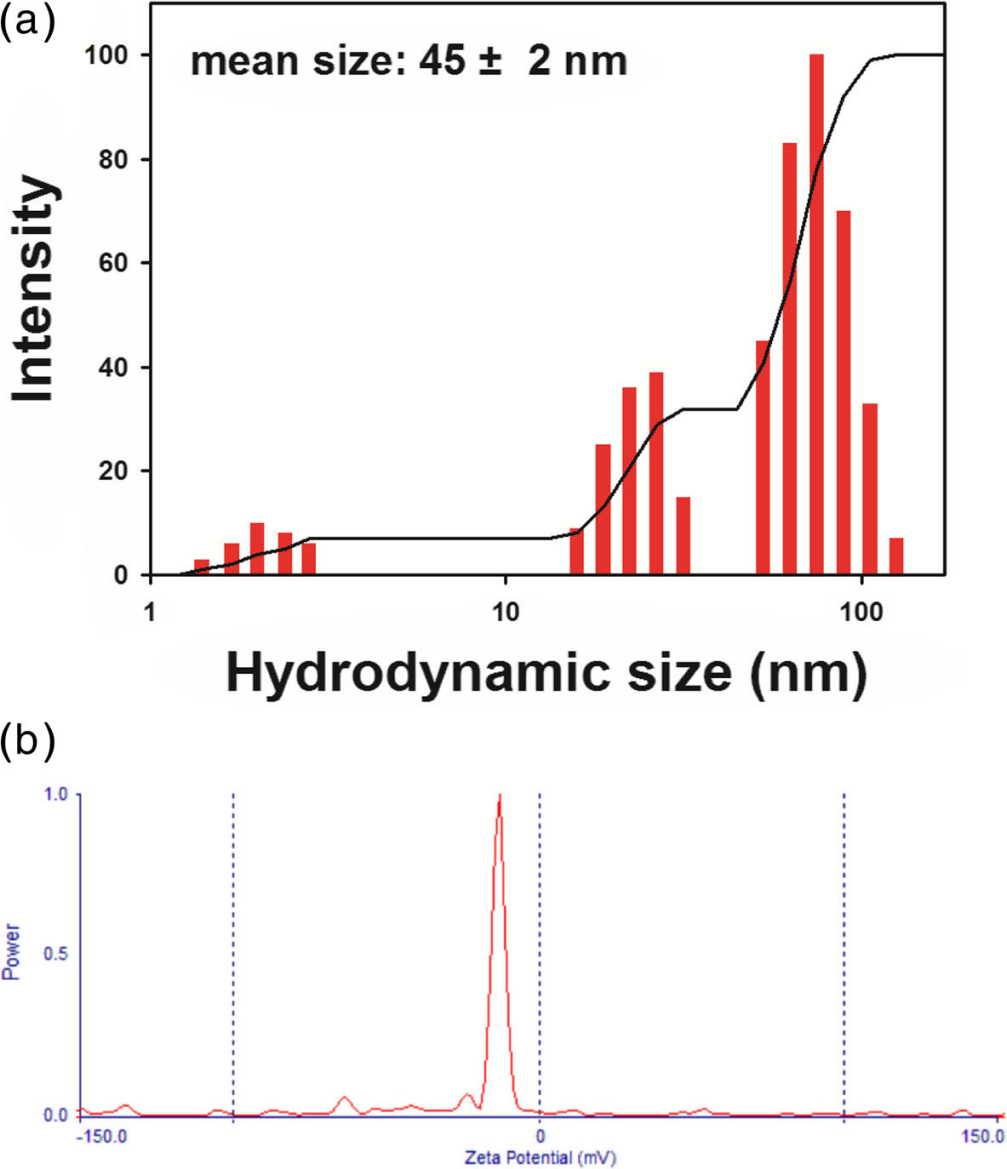
Hydrodynamic size and zeta potential of PV-AuNPs. **a** Hydrodynamic size. **b** Zeta potential

The hydrodynamic size and zeta potential are important characteristics in nanoparticle research for further applications. Generally, the size measured from HR-TEM images is smaller than that of the hydrodynamic size, as the hydrodynamic size reflects the biomolecules bound on the surface of the nanoparticles. As shown in Fig. 6a, the hydrodynamic size was determined to be 45 ± 2 nm with a polydispersity index of 0.258, which was larger than the size of the spheres measured from the HR-TEM images (23.8 ± 1.3 nm from 250 nanoparticles). This result clearly suggested that the phytochemicals in the extract bound to the surface of the PV-AuNPs and stabilized them. In addition, the PV-AuNPs possessed a negative zeta potential of − 13.99 mV (Fig. 6b). These results showed that phytochemicals with negative charges most likely contributed to the negative zeta potential. This negative zeta potential gives repulsive forces in a colloidal solution of PV-AuNPs endowing its stability. Therefore, one of our future works will include the phytochemical screening on the extract of *P. vulgaris* to elucidate the exact compounds which contribute to the negative zeta potential.

### HR-XRD Analysis of the PV-AuNPs

**Fig. 7:**
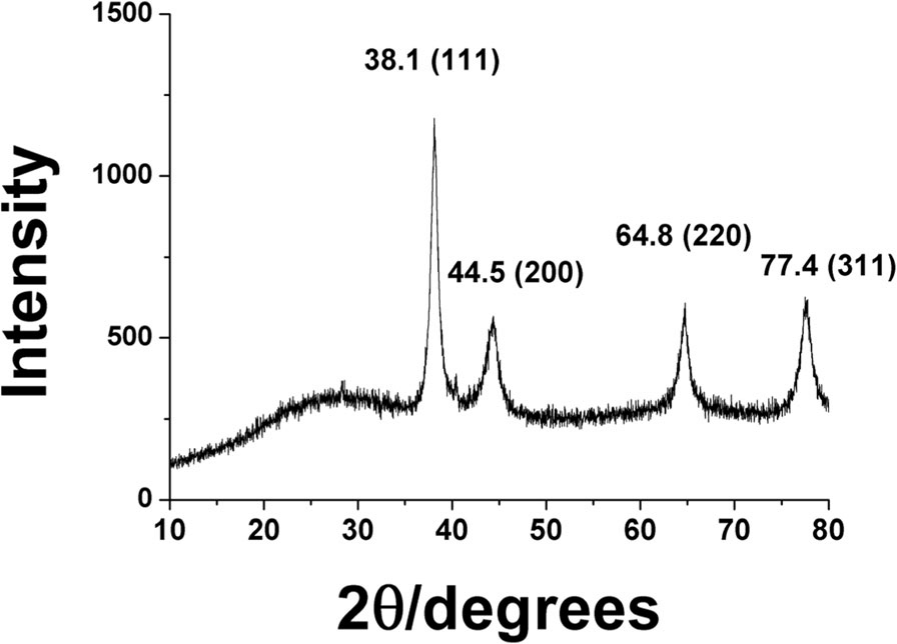
HR-XRD analysis of PV-AuNPs

X-ray diffraction analysis is generally employed to obtain crystallographic information about metallic nanoparticles. The characteristic diffraction patterns in terms of the Bragg reflections obtained from HR-XRD showed that the PV-AuNPs possessed a crystalline structure. The diffraction peaks (2*θ* values) observed at 38.1°, 44.5°, 64.8°, and 77.4° corresponded to the (111), (200), (220), and (311) planes, respectively, of a face-centered cubic structure in the PV-AuNPs (Fig. 7). The Scherrer equation (*D* = 0.89·λ/W·cos*θ*) was used to determine the approximate size of the PV-AuNPs. We selected the most intense (111) peak for application in the equation. The definition of each term in the equation is as follows: *D* is the size of the PV-AuNPs determined from the (111) peak, *θ* is the Bragg diffraction angle of the (111) peak, *λ* is the X-ray wavelength that was utilized, and W is the full width at half maximum (FWHM) of the (111) peak, in radians. From the Scherrer equation, the approximate size of the PV-AuNPs was determined to be 16.7 nm. The size determined from the Scherrer equation was smaller than the sizes measured from both HR-TEM images and the hydrodynamic size. The hydrodynamic size was the largest, possibly due to the fact that phytochemicals in the extract bound to the surface of the PV-AuNPs.

### Reaction Yield of the PV-AuNPs

ICP-OES was used to estimate the reaction yield. First, the total concentration of Au was obtained by analyzing the PV-AuNP solution. Second, the PV-AuNPs were centrifuged and the supernatant was collected. The supernatant was analyzed to ascertain the concentration of unreacted Au in the reduction reaction. Then, the reaction yield was estimated as follows: reaction yield (%) = 100 − [Au concentration in the supernatant/Au concentration in the colloidal solution] × 100.

Based on ICP-OES analysis, the concentrations of Au in the colloidal PV-AuNP solution and the supernatant were determined to be 96.337 ppm and 0.679 ppm, respectively. Therefore, the reaction yield of the PV-AuNPs was determined to be 99.3%. From the ICP-OES results, we concluded that the reaction condition was optimal which produced a high reaction yield of PV-AuNPs.

### Antioxidant Activity of the PV-AuNPs

**Fig. 8:**
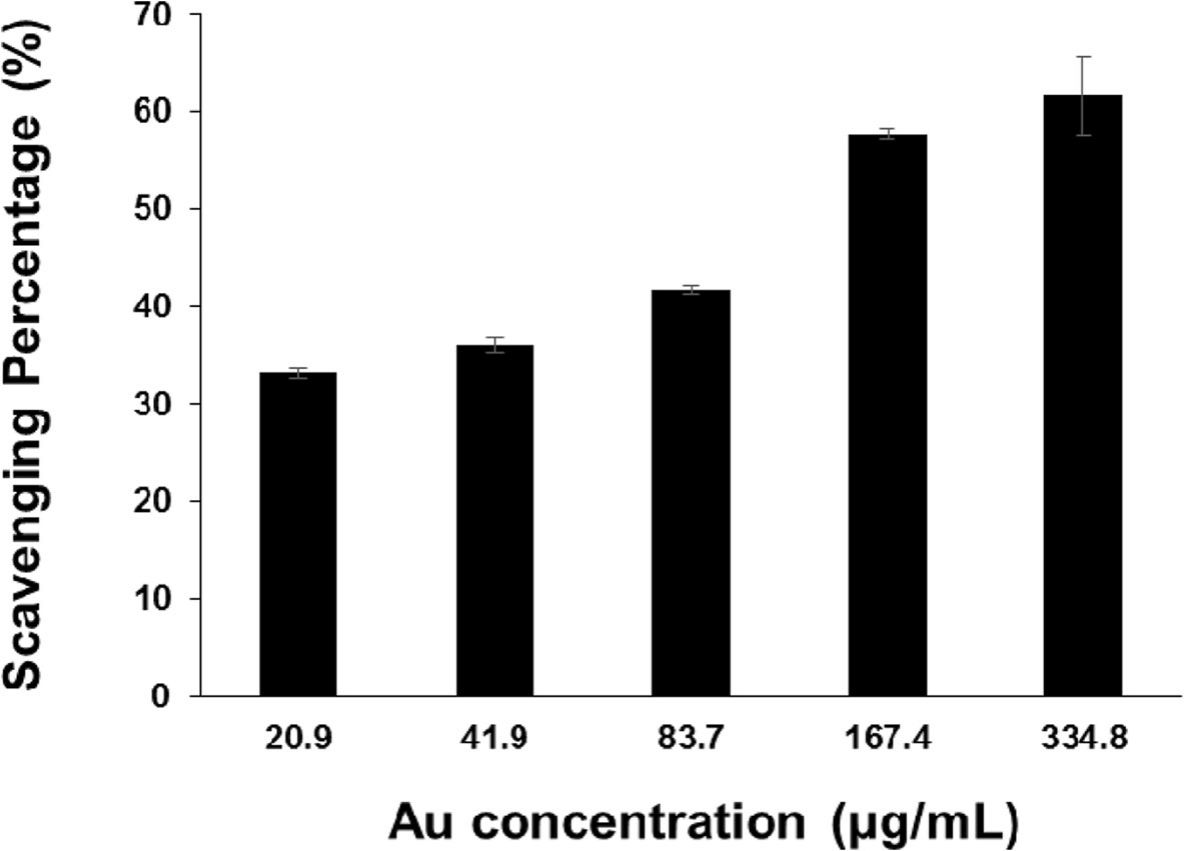
DPPH radical scavenging activity of PV-AuNPs. The concentration was expressed as Au concentration

The DPPH radical scavenging activity was examined to evaluate the antioxidant activity of the PV-AuNPs. As shown in Fig. 8, the DPPH radical scavenging activity of the PV-AuNPs was nearly constant at a concentration of less than 20.9 μg/mL (based on the Au concentration). However, as the concentration increased (41.9~334.8 μg/mL based on the Au concentration), the DPPH radical scavenging activity also increased, suggesting that the activity was dose-dependent. The IC_50_ of the PV-AuNPs was observed to be 165.0 μg/mL, which was equivalent to the IC_50_ of vitamin C (49.4 μM).

Green-synthesized AuNPs or AgNPs have been known to possess antioxidant activity in terms of DPPH radical scavenging activity [20, 21]. Ginseng-berry-mediated AuNPs demonstrated antioxidant activity [20]. The absorption of phytochemicals in the ginseng berry extract on the surface of nanoparticles having a large surface area can be credited to the antioxidant activity of AuNPs [20]. Green-synthesized AuNPs using *Angelica pubescens* roots showed antioxidant activity with a dose-dependent manner [21]. Secondary metabolites in *A. pubescens* such as flavonoids, sesquiterpenes, and phenolic compounds are potential contributors for the free radical scavenging activity [21]. However, AgNPs demonstrated higher antioxidant activity than AuNPs in the two articles mentioned above.

### Cytotoxicity of the PV-AuNPs

**Fig. 9:**
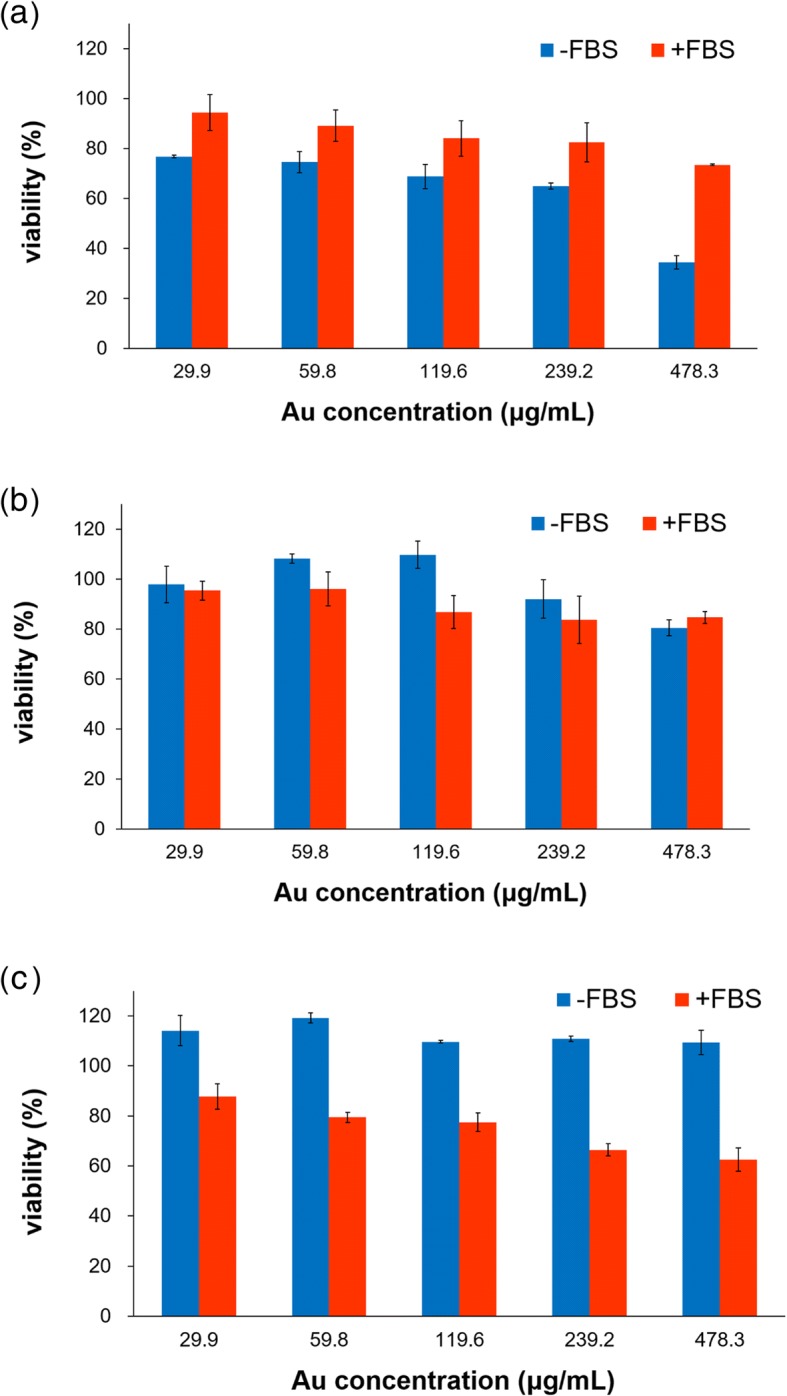
In vitro cytotoxicity on cancer cells. **a** HT-29. **b** MDA-MB-231. **c** PANC-1

The cytotoxicity of the PV-AuNPs against three cancer cells from different tissue types (colon, pancreas, and breast) was evaluated to examine the tissue-specific cytotoxicity of the nanoparticles in the presence/absence of FBS (Fig. 9). When serum was present during the cell culture, various kinds of protein coronas formed. The protein corona was dependent on the characteristics of the nanoparticles, such as the size, shape, surface charge, and surface roughness [22]. The protein corona can either increase or decrease the cellular uptake and cytotoxicity of the nanoparticles [23]. However, there are controversial reports regarding whether the protein corona causes the cytotoxicity to increase or decrease. Thus, the cytotoxicity of the PV-AuNPs was evaluated in the presence and absence of FBS. In the absence of FBS, HT-29 showed the greatest cytotoxicity at the tested concentrations (29.9~478.3 μg/mL) among three cells (Fig. 9a), followed by MDA-MB-231 (Fig. 9b) and lastly by PANC-1 (Fig. 9c). However, in the presence of FBS, these cells demonstrated a different phenomenon. PANC-1 showed the highest cytotoxicity, followed by HT-29 cells. In the absence of FBS and with 478.3 μg/mL Au, both the PANC-1 and MDA-MB-231 cells did not show any significant cytotoxicity, while strong cytotoxicity was observed for the HT-29 cells (65.6% cytotoxicity). However, in the presence of FBS, cytotoxicity was observed for PANC-1 (37.5% cytotoxicity) at 478.3 μg/mL Au. In general, the PV-AuNPs surrounded by a protein corona strongly influenced the cytotoxicity against cancer cells, although the results were tissue-specific. The protein corona produced by FBS increased the cytotoxicity against PANC-1 when compared with the cytotoxicity in the absence of FBS; however, the cytotoxicity decreased in the presence of the protein corona for HT-29 when compared with the cytotoxicity without the protein corona.

It has been reported that a variety of proteins bind to both positively and negatively charged AuNPs, while few proteins bind to AuNPs with a neutral charge [17, 24]. The negatively charged AuNPs showed a higher affinity and a slower release of fibrinogen protein than the positively charged AuNPs, suggesting the existence of specific binding sites for fibrinogen on the negatively charged AuNPs [17]. The PV-AuNPs possessed a negative zeta potential; thus, the binding of fibrinogen may affect the cytotoxicity against cancer cells. The investigation of the protein corona of nanoparticles is beneficial in nanomedicine research for future biomedical and clinical applications.

## Conclusions

The aqueous extracts of *A. capillaris*, *P. oleracea*, and *P. vulgaris* exhibited antioxidant activity which was evaluated by free radical scavenging activity, total phenolic content, and reducing power. *P. vulgaris* exerted the highest antioxidant activity, followed by *A. capillaris* and *P. oleracea*. The extract of *P. vulgaris* possessing the highest antioxidant activity was a very efficient green reducing agent for the biofabrication of AuNPs. The findings of our study demonstrate that the factors of the antioxidant activity, such as the free radical scavenging activity, reducing power, and total phenolic content, are closely correlated with the color of the colloidal solution and the shape of the SPR band of the resulting AuNPs. When the antioxidant activity was high, the resulting AuNPs showed a narrow SPR band with a strong absorbance. In contrast, a broad SPR band was observed for the PO-AuNPs, in which *P. oleracea* exhibited the lowest antioxidant activity among the three extracts. Furthermore, the PO-AuNPs aggregated easily after storage at ambient temperature. Consequently, the extract of *P. vulgaris* produced a wine-red colloidal solution of PV-AuNPs with various shapes with a maximum SPR of 530 nm. A face-centered cubic crystalline pattern of PV-AuNPs was confirmed by high-resolution X-ray diffraction analysis. The reaction yield was estimated by ICP-OES to be 99.3%. The hydrodynamic size (45 ± 2 nm) was larger than that measured from HR-TEM images suggesting that phytochemicals bound on the surface of the PV-AuNPs. Furthermore, phytochemicals with negative charges possibly attributed to a negative zeta potential of − 13.99 mV. The antioxidant activity of the PV-AuNPs was dependent on the Au concentration that was assessed based on the DPPH radical scavenging activity. Green-synthesized AuNPs using ginseng-berry and *A. pubescens* root extracts also showed DPPH radical scavenging activity [20, 21]. The PV-AuNPs showed cytotoxicity against HT-29, PANC-1, and MDA-MB-231 cells. Interestingly, the presence or absence of FBS dramatically affected the cytotoxicity against these cells. This phenomenon was possibly due to the protein corona that surrounded the surface of the nanoparticles.

Currently, AuNPs have diverse applications in nanomedicine as drug delivery vehicles or carriers of anticancer agents such as doxorubicin. *P. vulgaris* ethylacetate fraction showed cardioprotective effects with a concentration-dependent manner on isolated rat cardiomyocytes subjected to doxorubicin-induced oxidative stress [25]. PV-AuNPs can be applied as doxorubicin delivery vehicles with a cardioprotective ability. Therefore, our subsequent work will explore the anticancer activities of the PV-AuNPs loaded with doxorubicin for future biomedical and pharmaceutical applications. Diverse plant extracts have a potential to be effective reducing agents for biofabricating AuNPs. When the biofabrication reaction is conducted, it is essential to select a plant extract that possesses a high antioxidant activity in order to produce stable AuNPs. Additionally, *P. vulgaris* extract has a potential to be used as a reducing agent for the green synthesis of other novel metallic nanoparticles with valuable applications in the future.

## Abbreviations

ABTS: 2,2′-Azino-bis(3-ethylbenzothiazoline-6-sulfonic acid)
AC-AuNPs: Gold nanoparticles green synthesized by the extract of *A. capillaris*
AgNPs: Silver nanoparticles
AuNPs: Gold nanoparticles
BHT: Butylated hydroxytoluene
DMEM: Dulbecco’s modified Eagle’s medium
DPPH: 2,2-Diphenyl-1-picrylhydrazyl
FBS: Fetal bovine serum
GAE: Gallic acid equivalent
HR-TEM: High-resolution transmission electron microscopy
HR-XRD: High-resolution X-ray diffraction
HT-29: Human colorectal adenocarcinoma cell
IC_50_: Half maximal inhibitory concentration
ICP-OES: Inductively coupled plasma optical emission spectroscopy
MDA-MB-231: Human breast adenocarcinoma cell
PANC-1: Human pancreas ductal adenocarcinoma cell
PO-AuNPs: Gold nanoparticles green synthesized by the extract of *P. oleracea*
PV-AuNPs: Gold nanoparticles green synthesized by the extract of *P. vulgaris*
SPR: Surface plasmon resonance
WST assay: Water-soluble tetrazolium assay

## References

[1] Ramos AP, Cruz MAE, Tovani CB, Ciancaglini P (2017). Biomedical applications of nanotechnology. Biophys Rev.

[2] Jahangirian H, Lemraski EG, Webster TJ, Rafiee-Moghaddam R, Abdollahi Y (2017). A review of drug delivery systems based on nanotechnology and green chemistry: green nanomedicine. Int J Nanomedicine.

[3] Park Y, Hong YN, Weyers A, Kim YS, Linhardt RJ (2011). Polysaccharides and phytochemicals: a natural reservoir for the green synthesis of gold and silver nanoparticles. IET Nanobiotechnol.

[4] Bora Kundan Singh, Sharma Anupam (2010). The GenusArtemisia: A Comprehensive Review. Pharmaceutical Biology.

[5] Yang C, Hu DH, Feng Y (2015). Antibacterial activity and mode of action of the *Artemisia capillaris* essential oil and its constituents against respiratory tract infection-causing pathogens. Mol Med Rep.

[6] Kim KS, Yang HJ, Lee JY, Na YC, Kwon SY, Kim YC, Lee JH, Jang HJ (2014) Effects of *β*-sitosterol derived from *Artemisia capillaris* on the activated human hepatic stellate cells and dimethylnitrosamine-induced mouse liver fibrosis. BMC Complement Altern Med 14:363. 10.1186/1472-6882-14-363 10.1186/1472-6882-14-363 PMC4193130 25262005

[7] Lei X, Li J, Liu B, Zhang N, Liu H (2015). Separation and identification of four new compounds with antibacterial activity from *Portulaca oleracea* L. Molecules.

[8] Iranshahy M, Javadi B, Iranshahi M, Jahanbakhsh SP, Mahyari S, Hassani FV, Karimi G (2017). A review of traditional uses, phytochemistry and pharmacology of *Portulaca oleracea* L. J Ethnopharmacol.

[9] Oh C, Price J, Brindley MA, Widrlechner MP, Qu L, McCoy JA, Murphy P, Hauck C, Maury W (2011). Inhibition of HIV-1 infection by aqueous extracts of *Prunella vulgaris* L. Virol J.

[10] Feng Liang, Jia Xiao-Bin, Shi Feng, Chen Yan (2010). Identification of Two Polysaccharides from Prunella vulgaris L. and Evaluation on Their Anti-Lung Adenocarcinoma Activity. Molecules.

[11] Li C, Huang Q, Fu X, Yue XJ, Liu RH, You LJ (2015). Characterization, antioxidant and immunomodulatory activities of polysaccharides from *Prunella vulgaris* Linn. Int J Biol Macromol.

[12] Feng L, Jia X, Zhu MM, Chen Y, Shi F (2010). Antioxidant activities of total phenols of *Prunella vulgaris* L. in vitro and in tumor-bearing mice. Molecules.

[13] Raafat K, Wurglics M, Schubert-Zsilavecz M (2016). *Prunella vulgaris* L. active components and their hypoglycemic and antinociceptive effects in alloxan-induced diabetic mice. Biomed Pharmacother.

[14] Bai Yubing, Xia Bohou, Xie Wenjian, Zhou Yamin, Xie Jiachi, Li Hongquan, Liao Duanfang, Lin Limei, Li Chun (2016). Phytochemistry and pharmacological activities of the genus Prunella. Food Chemistry.

[15] Zanganeh S, Spitler R, Erfanzadeh M, Alkilany AM, Mahmoudi M (2016). Protein corona: opportunities and challenges. Int J Biochem Cell Biol.

[16] Shang L, Nienhaus GU (2016). Metal nanoclusters: protein corona formation and implications for biological applications. Int J Biochem Cell Biol.

[17] Vinluan RD, Zheng J (2015). Serum protein adsorption and excretion pathways of metal nanoparticles. Nanomedicine.

[18] Shannahan Jonathan H., Fritz Kristofer S., Raghavendra Achyut J., Podila Ramakrishna, Persaud Indushekar, Brown Jared M. (2016). From the Cover: Disease-Induced Disparities in Formation of the Nanoparticle-Biocorona and the Toxicological Consequences. Toxicological Sciences.

[19] Grade S, Eberhard J, Neumeister A, Wagener P, Winkel A, Stiesch M, Barcikowski S (2012). Serum albumin reduces the antibacterial and cytotoxic effects of hydrogel-embedded colloidal silver nanoparticles. RSC Adv.

[20] Jiménez Pérez ZE, Mathiyalagan R, Markus J, Kim YJ, Kang HM, Abbai R, Seo KH, Wang D, Soshnikova V, Yang DC (2017). Ginseng-berry-mediated gold and silver nanoparticle synthesis and evaluation of their in vitro antioxidant, antimicrobial, and cytotoxicity effects on human dermal fibroblast and murine melanoma skin cell lines. Int J Nanomedicine.

[21] Markus J, Wang D, Kim YJ, Ahn S, Mathiyalagan R, Wang C, Yang DC (2017). Biosynthesis, characterization, and bioactivities evaluation of silver and gold nanoparticles mediated by the roots of Chinese herbal *Angelica pubescens* Maxim. Nanoscale Res Lett.

[22] Kettler K, Giannakou C, de Jong WH, Hendriks AJ, Krystek P (2016). Uptake of silver nanoparticles by monocytic THP-1 cells depends on particle size and presence of serum proteins. J Nanopart Res.

[23] Corbo C, Molinaro R, Parodi A, Toledano Furman NE, Salvatore F, Tasciotti E (2016). The impact of nanoparticle protein corona on cytotoxicity, immunotoxicity and target drug delivery. Nanomedicine.

[24] Bodelón G, Costas C, Pérez-Juste J, Pastoriza-Santos I, Liz-Marzán LM (2017). Gold nanoparticles for regulation of cell function and behavior. Nano Today.

[25] Psotova J, Chlopcikova S, Miketova P, Simanek V (2005). Cytoprotectivity of *Prunella vulgaris* on doxorubicin-treated rat cardiomyocytes. Fitoterapia.

